# Pangenome and genomic taxonomy analyses of *Leuconostoc gelidum* and *Leuconostoc gasicomitatum*

**DOI:** 10.1186/s12864-022-09032-3

**Published:** 2022-12-09

**Authors:** Per Johansson, Elina Säde, Jenni Hultman, Petri Auvinen, Johanna Björkroth

**Affiliations:** 1 Department of Food Hygiene and Environmental Health, University of Helsinki, Helsinki, Finland; 2 Institute of Biotechnology, University of Helsinki, Helsinki, Finland

**Keywords:** Lactic acid bacteria, Genomic taxonomy, Pangenome, Phylogeny, *Leuconostoc*

## Abstract

**Background:**

*Leuconostoc gelidum* and *Leuconostoc gasicomitatum* have dual roles in foods. They may spoil cold-stored packaged foods but can also be beneficial in kimchi fermentation. The impact in food science as well as the limited number of publicly available genomes prompted us to create pangenomes and perform genomic taxonomy analyses starting from de novo sequencing of the genomes of 37 *L. gelidum/L. gasicomitatum* strains from our culture collection. Our aim was also to evaluate the recently proposed change in taxonomy as well as to study the genomes of strains with different lifestyles in foods.

**Methods:**

We selected as diverse a set of strains as possible in terms of sources, previous genotyping results and geographical distribution, and included also 10 publicly available genomes in our analyses. We studied genomic taxonomy using pairwise average nucleotide identity (ANI) and calculation of digital DNA-DNA hybridisation (dDDH) scores. Phylogeny analyses were done using the core gene set of 1141 single-copy genes and a set of housekeeping genes commonly used for lactic acid bacteria. In addition, the pangenome and core genome sizes as well as some properties, such as acquired antimicrobial resistance (AMR), important due to the growth in foods, were analysed.

**Results:**

Genome relatedness indices and phylogenetic analyses supported the recently suggested classification that restores the taxonomic position of *L. gelidum* subsp. *gasicomitatum* back to the species level as *L. gasicomitatum*. Genome properties, such as size and coding potential, revealed limited intraspecies variation and showed no attribution to the source of isolation. The distribution of the unique genes between species and subspecies was not associated with the previously documented lifestyle in foods. None of the strains carried any acquired AMR genes or genes associated with any known form of virulence.

**Conclusion:**

Genome-wide examination of strains confirms that the proposition to restore the taxonomic position of *L. gasicomitatum* is justified. It further confirms that the distribution and lifestyle of *L. gelidum* and *L. gasicomitatum* in foods have not been driven by the evolution of functional and phylogenetic diversification detectable at the genome level.

**Supplementary Information:**

The online version contains supplementary material available at 10.1186/s12864-022-09032-3.

## Background

*Leuconostoc gelidum* is a psychrotrophic lactic acid bacterium (LAB) detected by Shaw and Harding [1] in cold-stored, vacuum-packaged beef. In 2014, *Leuconostoc gasicomitatum* [2] was reclassified as *Leuconostoc gelidum* subsp. *gasicomitatum* [3] and another subspecies, *Leuconostoc gelidum* subsp. *aenigmaticum* was described. Reclassification of *L. gasicomitatum* was mainly based on the results from the phylogenetic analyses of *atpA*, *pheS*, and *rpoA* housekeeping genes. The sequence analyses of concatenated *atpA*, *pheS*, and *rpoA* genes had demonstrated that the novel strains, designated as subsp. *aenigmaticum*, as well as the type and reference strains of *L. gelidum* and *L. gasicomitatum,* were phylogenetically closely related. Until recently, *L. gelidum* was thus considered to comprise three subspecies i.e., subsp. *gelidum*, *gasicomitatum* and *aenigmaticum*, but based on the ANI and dDDH values of *L. gelidum* type strains, Wu and Gu [4] proposed to reject the proposal of Rahkila et al. [3], and to restore the taxonomic position of *L. gelidum* subsp. *gasicomitatum* back to the species level as *L. gasicomitatum.*

*L. gelidum* and *L. gasicomitatum* have commonly been associated with the spoilage of perishable food items, such as meat and poultry or minimally processed vegetables [5–7]. Metagenomic analyses have detected these species mainly in cold-stored meat and vegetables [8], but rarely in samples from animal microbiomes or environmental sources. Apart from food spoilage, *L. gelidum* and *L. gasicomitatum* have often been associated with the fermentation of kimchi [9, 10], a Korean traditional side dish made most commonly of Chinese cabbage and Korean radishes with a variety of seasonings. Kimchi is often fermented at low temperature (2–6 °C) to restrict the growth of spoilage bacteria, but psychrotrophic LAB, *L. gelidum* and *L. gasicomitatum* can grow at temperatures below 6 °C [1, 3, 6]. Therefore, they may become abundant in kimchi microbiomes [9–11]. Thus, the growth of *L. gelidum* in refrigerated foods may either be unwanted and lead to spoilage or be endorsed to ensure proper ripening and preservation according to the expectations of kimchi fermentation.

The significance of *L. gelidum* and *L. gasicomitatum* in food, prompted our interest to further studying these species at the genome level. Despite the importance of these species in the food system, relatively little is known about them at the genome level, and at the time of this study, only few genomes were available in the public databases. Until now, most studies have applied analysis of molecular fingerprints [6, 7] or multi-locus sequence typing [12] to examine these species. In addition, the recent taxonomic proposal warranted further genotaxonomic studies performed with a larger set of strains than just the type strains that were used by Wu and Gu [4]. Since we have worked with these species during the course of two decades, our strain collection allowed us to select a diverse set of well-characterised strains in terms of sources, previously conducted genotyping results and geographical distribution (Supplementary Table S1) for the present study.

## Results

### Genome features reveal little intraspecies variation

The 37 *L. gelidum/L. gasicomitatum* strains (our *L. gelium* subsp. *gasicomitatum* strains were renominated as *L. gasicomitatum* in this paper according to the proposal of Wu and Gu [4]) had an average genome size of 1.95 Mbp (range 1.82–2.12 Mbp) with GC content ranging from 36.4 to 36.8% (Table 1, Supplementary Table S1). The number of contigs ranged from 6 to 49 (av. 24 ± 12; Table 1) with 28 of 37 (75%) of the genomes that were assembled to a maximum of 32 contigs. The N50 values ranged from 102 kbp to 1.45 Mbp (Table 1), with an average of 354 kbp (Supplementary Table S2). On average, the genomes encoded 1,842 predicted proteins (range 1,704–2,034). The number of pseudogenes was estimated to range from 33 to 67 (43 ± 7.6). Furthermore, the number of transfer RNA (tRNA) genes varied from 40 to 47 with an average of 44 tRNA genes per assembled genome. (Supplementary Table S2).

**Table 1:** *Leuconostoc* strains (*n* = 37) sequenced and genomic features of the draft genomes

Strain	Source, country	Size (Mbp)	GC (%)	Contigs	N50	CDS	tRNA
*L. gasicomitatum*							
10.16.3	Processing plant, Belgium	2.02	36.5	32	121027	1952	44
6.2.3	Processing plant, Belgium	2.02	36.6	29	216693	1957	44
A.21.4	Iceberg lettuce, Belgium	1.94	36.6	21	159428	1841	46
A.5.3	Celery, Belgium	2.05	36.6	47	157345	1961	44
A.8.4	Cucumber, Belgium	1.99	36.5	30	213388	1897	44
ab.2	Acid bath, Belgium	2.00	36.5	24	182261	1890	44
EPV3	Pork, Spain	1.97	36.5	12	1176892	1855	43
HS1	Boiled egg, Belgium	2.00	36.5	44	134579	1924	43
HS10	Boiled egg, Belgium	2.00	36.5	44	144646	1922	43
JL3-1	Minced meat, Finland	1.91	36.6	34	126334	1804	44
Jla4-8	Minced meat, Latvia	1.91	36.6	17	231914	1810	43
JP13-3	Sausage plant, Finland	2.01	36.6	15	326528	1878	46
Mkl1-2	Marinated fish, Finland	1.91	36.7	13	415607	1788	43
Mkl2-18	Marinated fish, Finland	1.90	36.7	20	193248	1794	44
Ms25-3	Broiler meat, Finland	1.91	36.4	33	108553	1822	40
NAFIM5a-6	Marinated beef, Finland	1.87	36.8	28	101644	1766	42
POHU19	Pork, Finland	1.90	36.8	25	147848	1801	45
POULM2-8	Marinated pork, Finland	1.95	36.8	49	126244	1845	47
R-46608	Blood sausage, Belgium	2.12	36.5	47	211750	2034	43
R-46710	Salad blend, Belgium	2.02	36.6	23	157421	1932	44
R-46850	Salad blend, Belgium	1.98	36.6	33	191247	1895	43
R-46920	Bell pepper, Belgium	1.94	36.6	32	153227	1854	42
RSNU1f	Beef, Finland	1.85	36.8	27	147651	1736	46
Vvan8	Beef, Finland	1.82	36.7	27	147706	1734	46
*L. gelidum*							
subsp. *aenigmaticum*							
DSM 19374	Broiler meat, Finland	1.95	36.6	11	481315	1834	43
DSM 19375^T^	Pork, Finland	1.94	36.6	10	1445491	1806	46
POKY4-4	Pork, Finland	1.93	36.6	12	399502	1811	43
subsp. *gelidum*							
AMKR21	Broiler leg, Finland	1.91	36.7	10	633552	1790	44
C220d	Salad blend, Finland	1.97	36.6	24	186934	1847	44
Ebr1-8	Broiler fillet, Spain	1.92	36.8	6	1151147	1820	42
HS9	Cooked ham, Finland	1.90	36.7	10	599704	1765	42
JPBL22	Sausage plant, Finland	1.89	36.7	10	559305	1762	45
KAPA3-9	Meat, Finland	1.92	36.7	11	1053052	1812	45
Kg1-2	Vegetarian sausage, Finland	1.83	36.7	9	559672	1704	41
PB4d	Carrot, Finland	2.01	36.6	26	126988	1884	42
PLK1c	Carrot, Finland	1.92	36.7	11	378153	1785	46
Vvan9	Beef, Finland	1.97	36.6	32	179892	1854	45

*bp* Base pairs, *CDS* Coding sequences

### Genomic relatedness indices suggest species status for *L. gelidum* subsp. *gasicomitatum*

Pairwise average nucleotide identity (ANI) values were used to assess inter- and intra-subspecies relatedness. The values for *L. gelidum* subsp. *aenigmaticum* assemblies against *L. gelidum* subsp. *gelidum* assemblies (Table 2;Supplementary Table S4) were above 95%, the cut-off value for species delineation [13], which supports their classification to the same species. In contrast, *L. gasicomitatum* and *L. gelidum* subsp. *gelidum* DSM 5578^ T^ type assembly or *L. gelidum* subsp. *aenigmaticum* DSM 19375^ T^ type assembly presented ANI ≤ 94.8% (Table 2; Supplementary Table S4), and were, thus, lower than the proposed species cut-off values for ANI. Similarly, the average values for *L. gasicomitatum* against to the assemblies of subsp. *gelidum* and subsp. *aenigmaticum* were below the cut-off of 95% (Table 2); although 5% (21 of 416) of the pairwise comparisons were between 95.0–95.2% (Supplementary Table S4). The ANI values obtained for all the intra-subspecies comparisons between *L. gelidum* assemblies were high, i.e. ≥ 98% (Table 2, Supplementary Table S4).

**Table 2:** Pairwise average nucleotide identity (ANI) and dDDH digital DNA-DNA hybridisation (dDDH) values determined

Genome	*L. gelidum* subsp. *gelidum* DSM 5578^T^		*L. gelidum* subsp. *aenigmaticum* DSM 19375^T^		*L. gasicomitatum* LMG 18811^T^	
Method	ANI	dDDH	ANI	dDDH	ANI	dDDH
LMG 18811^T^	94.2	56.4	94.5	59.4	*	*
6.2.3	94.0	55.9	94.4	58.9	99.7	98.5
10.16.3	94.1	56.1	94.4	59.1	99.6	97.9
A.21.4	94.0	55.7	94.6	58.7	99.2	94.1
A.5.3	94.4	57.0	94.6	59.4	98.7	90.0
A.8.4	94.3	57.8	94.7	59.3	98.8	89.7
ab.2	94.5	58.2	94.8	59.6	98.7	91.6
C120C	94.1	56.3	94.6	58.9	99.6	99.0
C-122c	94.0	55.4	94.4	58.4	99.4	96.2
EPV3	94.0	55.7	94.6	58.9	99.7	98.4
HS1	94.3	57.2	94.5	59.6	98.8	91.9
HS10	94.3	57.2	94.4	59.7	98.8	92.0
JL3-1	94.0	55.8	94.3	58.6	98.7	91.7
Jla4-8	94.2	56.3	94.5	59.0	99.0	92.9
JP13-3	94.5	57.8	94.8	60.1	98.8	92.6
Kg16-1	94.1	56.0	94.6	59.5	99.1	94.1
KSL4-2	94.1	56.9	94.6	59.2	99.8	98.7
MKL1-2	94.4	57.8	94.6	59.3	98.6	89.7
MKL2-18	93.9	55.5	94.2	58.4	98.6	91.5
MS25-3	94.0	55.7	94.4	58.9	99.4	98.7
Nafim5a-6	94.3	57.1	94.5	59.6	98.8	91.6
PB1a	94.7	59.2	94.7	60.4	99.1	93.9
PB1e	94.0	55.5	94.5	59.0	99.0	92.7
PL111	94.2	56.0	94.5	59.1	99.7	98.9
POHU19	94.2	56.2	94.4	59.3	98.6	92.5
POULM2-8	93.9	55.4	94.1	58.6	98.4	91.7
R-46608	94.1	55.9	94.5	58.9	99.7	98.7
R-46710	94.3	56.6	94.4	59.5	98.7	89.0
R-46850	94.0	55.6	94.6	59.1	99.8	98.7
R-46920	94.0	55.7	94.7	59.1	99.4	96.1
RSNU1f	93.9	56.2	94.5	59.6	98.9	92.7
Vvan8	94.0	56.2	94.4	59.3	98.9	92.5
*Range of values*	93.9–94.7	55.4–59.2	94.1–94.8	58.4–60.4	98.4–99.8	89.0–99.0

In addition to ANI, we assessed the genome relatedness by calculating the pairwise digital DNA-DNA hybridisation (dDDH) scores for the 47 genomes (Table 2, Supplementary Table S4). For dDDH, similarity values ≥ 70% are considered an argument to classify the strains to the same species [14]. Similarly, with ANI metrics, *L. gasicomitatum* assemblies showed relatively low dDDH values against *L. gelidum* subsp. *gelidum* (57.7 ± 1.7%) and *L. gelidum* subsp. *aenigmaticum* (59.2 ± 0.5%) assemblies. The inter-subspecies values obtained for *L. gelidum* subsp. *aenigmaticum* against *L. gelidum* subsp. *gelidum* genomes varied more. They ranged from 67.1 to 87.4 (71.1 ± 4.6%) with the values obtained for the *L. gelidum* subsp. *gelidum* DSM 5578^T^ type assembly (69.0–72.4%) thus being also around the species cut-off of 70% (Table S4). However, the dDDH similarity was 70.6% between the type assemblies of these subspecies (DSM 5578^T^ × DSM 19275^T^) (Supplementary Table S4).

### Phylogenetic analyses classified subsp. *gasicomitatum* separately from subs. *gelidum*

**Fig. 1:**
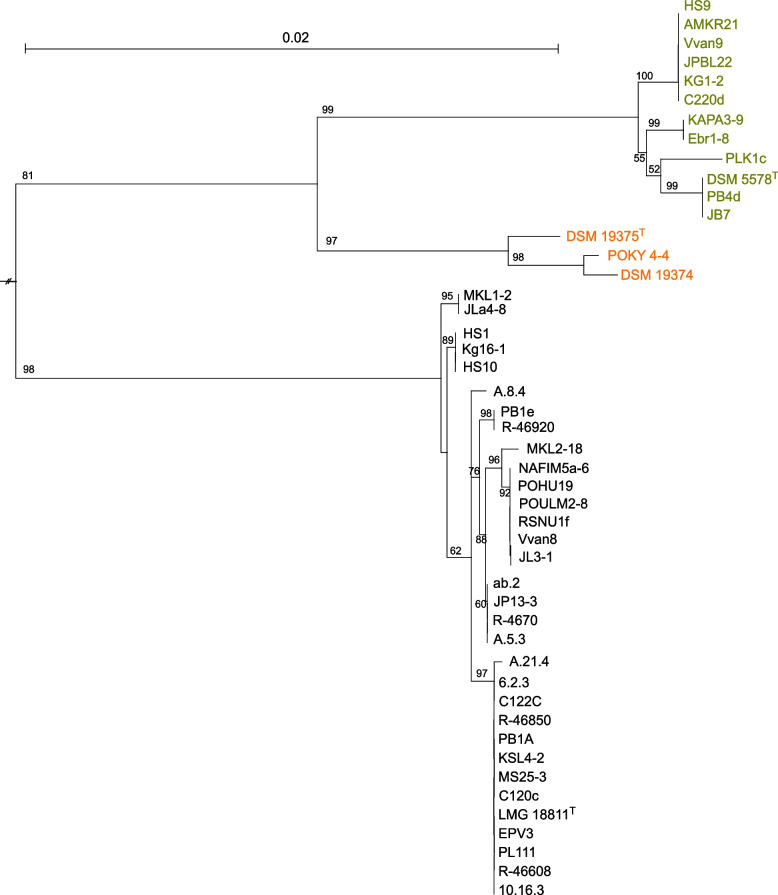
Maximum-Likelihood phylogenetic tree for concatenated housekeeping genes *atpA*, *pheS* and *rpoA* nucleotide sequences. Bootstrap support (%) for 1,000 replicates are given at branch points

**Fig. 2:**
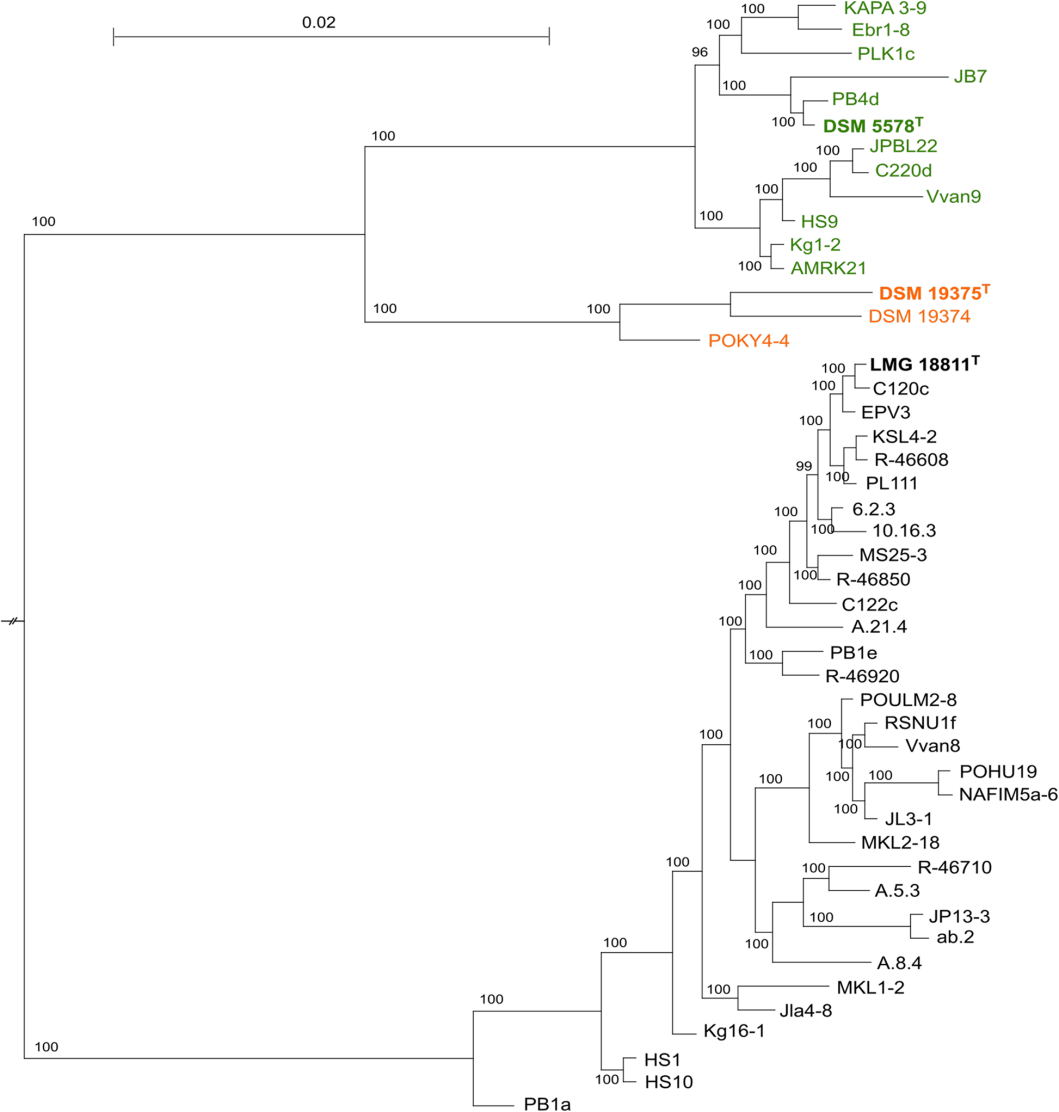
Maximum-Likelihood phylogenetic tree for 1,141 single-copy orthologous genes shared by all genomes. Bootstrap support (%) for 1,000 replicates are given at branch points

To assess the evolutionary relationships between the strains belonging to the former three *L. gelidum* subspecies, we constructed a maximum likelihood phylogenetic tree (Fig. 1) from concatenated nucleotide sequences of three housekeeping genes *atpA, pheS* and *rpoA* used in the previous taxonomy studies to distinguish *Leuconostoc* species [3, 15]. In the tree topology (Fig. 1), the *L. gasicomitatum* strains were separated with high bootstrap support from subsp. *gelidum* and subsp. *aenigmaticum* strains. For this finding, we obtained even stronger support with a tree constructed using a core gene set of 1,141 single-copy genes (Fig. 2). In both phylogenetic treeing approaches, the three subsp. *aenigmaticum* strains clustered together and were placed in the same group as the subsp. *gelidum* strains (Figs. 1 and 2). In addition, we did not notice any clustering based on the geographical locations of the *L. gasicomitatum* strains. Strains from Belgium (strains coded with R-, HS- and a- or numbers only) and Latvia (Jla4-8) clustered among those isolated in Finland.

### Pangenome analyses

**Fig. 3:**
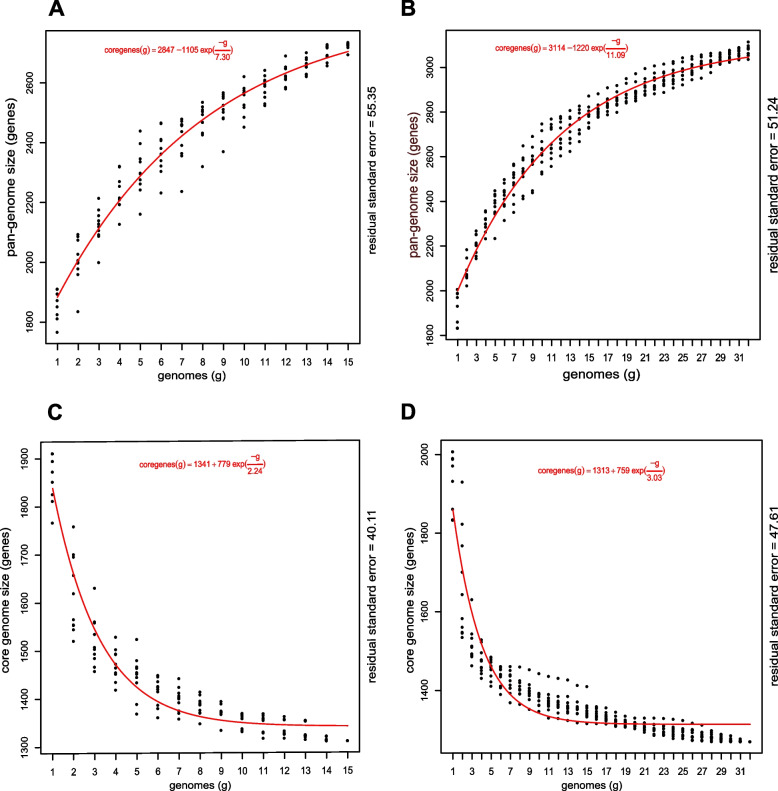
Estimates after 10 random genomes for pangenome (**A**) and core genome (**C**) for 15 *Leuconostoc gelidum* subsp. *gelidum* and *L. gelidum* subsp. *aenigmaticum* genomes, and for pangenome (**B**) and core genomes (**D**) for 32 *L. gasicomitatum* genomes. Curve fitted using the Tettelin model [16]. Residual standard errors reported to show the goodness of fit

We estimated the pangenome and core genome sizes separately for *L. gasicomitatum* and for *L. gelidum* subsp. *gelidum* and subsp. *aenigmaticum* using the model of Tettelin et al. [16] for curve fitting*.* Gene accumulation curves (Figs. 3 A and B) showed that the number of new genes added by each assembly continued to increase, indicating open pangenomes for both analyses. However, the expansion of the *L. gasicomitatum* pangenome was decreasing (Fig. 3B), and after 22 genomes fewer than 15 genes were added to the pool per new genome, suggesting that the pangenome was closing slowly. The core genome analyses (Figs. 3C and D) fitted the exponential decay model. The fitted curve for *L. gasicomitatum* plateaued at around 1300 genes (Fig. 3D), suggesting relatively stable core genomes.

The pangenome of *L. gasicomitatum* was estimated to include 3,046 genes of which 1,313 (43.1%) were considered the core genes present in 99% of the assemblies. On average, the core genome consisted of 71% (64–75%) of the coding potential of a *gasicomitatum* assembly. The open pangenome for *L. gelidum* subsp. *gelidum* and *aenigmaticum* consisted of 2703, gene, and of these 1,341 were included in the core genome. The unique genes detected in *L. gelidum*/*L. gasicomitatum* (Supplementary tables S5-S7) did not encode any properties associated with specific lifestyles related to food spoilage. Genes unique to *L. gelidum* subsp. *gelidum* and *L. gasicomitatum* strains were not detected.

### Properties associated with lifestyle in food

Regarding to properties typical for leuconostocs, all strains contained genes encoding at least one Glucansucrase and half of them carried also genes encoding a fructansucrase. No genes associated with the formation of biogenic amines through amino acid decarboxylation or deamination were found.

*L. gelidum* and *L. gasicomitatum* are known to be able to cause spoilage of food products by producing yellow carotenoids when growing in food that contains fat, e.g. ham or sausage [17, 18]. But the genes responsible for carotenoid production in LAB (including other *Leuconostoc* species), e.g. *crtN* and *crtM* [19], were not detected in *L. gelidum*/*L. gasicomitatum*. It is, therefore, not clear to us how *L. gelidum* and *L. gasicomitatum* produce carotenoids or what type of carotenoids they produce.

### Genetic determinants for fosfomycin and fusidic acid resistance

Next, we analysed the acquired antimicrobial resistance (AMR) determinants by comparing the *L. gelidum/ L. gasicomitatum* assemblies with the Comprehensive Antibiotic Resistance Database (CARD). The searches did not return any hits with good support (using “Perfect, Strict, complete genes only” as criteria) suggesting that these food-associated *L. gelidum/L. gasicomitatum* strains do not carry acquired or transferable antimicrobial resistance genes or allelic variants that cause antimicrobial resistance.

Regarding intrinsic resistance, the genus *Leuconostoc* is known to be resistant to vancomycin and the resistance mechanism is well-characterised [20]. Consistently, all 37 newly sequenced *L. gelidum* genomes harboured a resistant genotype of *ddl* gene. In addition to vancomycin, resistance to fosfomycin is common among leuconostocs [21, 22], and so we analysed the genomes for genes associated with fosfomycin resistance [23]. Among the alignments, the only known resistance determinant we identified was an amino acid substation in the fosfomycin target gene *murA*. A multiple protein sequence alignment of the MurA demonstrated (Supplementary Fig. 1) that all 47 *L. gelidum* strains had Asp substitution at position 115 (*E. coli* numbering) at the site encoding the active site of the MurA*,* a substitution known to confer fosfomycin resistance in *E. coli* [24].

Furthermore, we assessed the known genetic determinants behind fusidic acid resistance, another resistance characteristics of leuconostocs [25]. Sequence analysis of *fusA* (Supplementary Fig. 2)*,* a chromosomal gene encoding elongation factor G (EF-G), the target protein of fusidic acid, revealed alterations that cause resistance-associated amino acid substitution in EF-G [26]. Comparison with *Staphylococcus aureus* showed (Supplementary Fig. 2) that *L. gelidum/L. gasicomitatum* genomes harboured an amino acid substitution V90I, His457Q, Leu461M and S416T (*S. aureus* numbering) of which at least V90I and His457Q result in high fusidic acid resistance in *S. aureus* [26].

## Discussion

The genomic features of the 37 *L. gelidum/L. gasicomitatum* strains sequenced in the present study showed only little variation (Table 1; Supplementary Table S1), even though we selected as heterogenous a set of strains as possible from our database. We used MLST, fingerprinting and source attribution data accumulated over the past 25 years since 1995 (Table 1; Supplementary Table S1) to select the strains for the present study. The genome sizes (1.95 ± 0.06 Mbp), GC contents (36.6 ± 0.1%), numbers of CDS (1842 ± 72) and tRNA genes (44 ± 2) were quite similar (Table 1; Supplementary Table S2) and alike with the features present in the genomes of the 10 *L. gelidum/L. gasicomitatum* strains (Table 1; Supplementary Table S3) available in the public NCBI database.

The genetic relatedness indices (Table 2; Supplementary Table S4) as well as the phylogenetic analyses (Figs. 1 and 2) revealed that the strains formerly designated as *L. gelidum* subsp. *gasicomitatum* formed a genetically coherent group that was evolutionary different from the group containing the strains of the two other *L. gelidum* subspecies. According to the ANI and dDDH results (Table 2 and Supplementary Table 4), the taxonomic position of subsp. *gasicomitatum* within *L. gelidum* is not supported. Based on the ANI and dDDH results between the type strains of *L. gelidum* subspecies, Gu and Wu [4] have already suggested rejecting the proposal of Rahkila et al. [3] to reclassifiy *L. gasicomitatum* as *L. gelidum* subsp. *gasicomitatum*. Our study conducted with these 37 strains supports their findings and thus, the strains previously considered as subsp. *gasicomitatum* should be designated as *L. gasicomitatum*.

Rahkila et al. [3] justified the splitting of *L. gelidum* between three subspecies mainly because of the results obtained in the phylogenetic and optical DDH analyses. At that time, the phylogenic analyses were targeted at three housekeeping genes *atpA*, *pheS* and *rpoA* that had been reported to distinguish LAB species well [15]. Rahkila et al. [3] used 13 strains consisting of 6, 3 and 4 representatives of *L. gelidum* subsp. *aenigmaticum*, *L. gelidum* subsp. *gelidum* and *L*. *gasicomitatum*, respectively. Since the analysis of the concatenated *atpA*, *pheS* and *rpoA* sequences divided these 13 strains between three separate clusters, they subsequently conducted the optical DDH experiments using representative strains from each three clusters. In these DDH experiments, Rahkila et al. [3], used, among others, the type strains LMG18297^T^ and LMG18811^T^ of *L. gelidum* and *L. gasicomitatum*, respectively, and POUF4d, which is the current type strain (DSM 19375^T^) of subsp. *aenigmaticum*. The DDH result of 75% between *L. gelidum* and *L. gasicomitatum* type strains that they reported contravened the values previously published in two other studies. Björkroth et al. [2] and Kim et al. [27] had reported DDH values below 70%, i.e., 22 and 6%, respectively. In addition, Björkroth et al. (2000) had reported another value below 70%, i.e., 34%, between *L. gasicomitatum* LMG 18812 and *L. gelidum* subsp. *gelidum* LMG 9850^T^ type stain. Thus, the results of the present study are in accordance with the conclusions of Björkroth et al. (2000), Kim et al. (2000) and Gu and Wu (2021), supporting that the taxonomic position of *L. gelidum* subsp. *gasicomitatum* should be restored as *L. gasicomitatum*.

Our analyses also show that subsp. *aenigmaticum* has been correctly classified as a subspecies of *L. gelidum*. During the past 25 years, we have isolated and constructed a comprehensive culture collection containing hundreds of *L. gelidum* and *L. gasicomitatum* food isolates. However, we have not detected more subsp. *aenigmaticum* strains than those used in the study of Rahkila et al. [3]. To our knowledge, only one additional scientific study of this subspecies has been published thus far. Mun et al. (2021) found *L. gelidum* subsp. *aenigmaticum* LS4 strain to improve the organoleptic qualities of kimchi juice and suggested that LS4 could be used as a functional starter culture for food (vegetable or fruit) fermentation at low temperatures. The genome and functional properties of LS4 are very similar to those detected in this study and by Rahkila et al. [3]. The reason for the low detection rate of subsp. *aenigmaticum* is not known to us, but the genetic differences between subsp. *aenigmaticum* and *gelidum*, and even between *L. gasicomitatum*, do not explain any specific traits to suggest reasons for the rare detection (Supplementary Table S5).

Figures 2 and 3 present phylogenetic trees generated with the concatenated *atpA*, *pheS* and *rpoA* and WG sequences, respectively. Both approaches resulted in trees containing three separate clusters with either *L. gelidum* subsp. *gelidum*, *L. gelidum* subsp. *aenigmaticum* or *L. gasicomitatum* strains. In both trees *L. gelidum* subsp. *gelidum* and *L. gelidum* subsp. *aenigmaticum* strains clustered adjacently, whereas *L. gasicomitatum* strains were located more distantly from the other two clusters. This shows that *L. gelidum* and *L. gasicomitatum* differ clearly from each other also phylogenically. No clustering to suggest any attribution to specific isolation sources was found as in the previous *L. gasicomitatum* MLST study by Rahkila et al. [12].

Antimicrobial resistance in *Leuconostoc* is of interest to the researcher community since leuconoctocs are present in various foods [6, 7, 11, 12, 22] and antimicrobial resistance, particularly, transferable or acquired resistance-related determinants, is considered a food safety concern [28]. Antimicrobial resistance properties can also be used to develop tools for a counterselection marker for recombination [29] or to design differentiating or selecting growth media for LAB [25]. Leuconostocs are Generally Regarded As Safe (GRAS, US Food and Drug Administration); or some have the Qualified Presumption of Safety by the European Food Safety Authority. However, mainly because of their intrinsic antimicrobial vancomycin resistance, they have also been associated with infections in patients receiving vancomycin. According to our analyses of the 47 *L. gelidum/L. gasicomitatum* genomes, these strains did not carry any acquired antimicrobial resistance genes. In addition to intrinsic vancomycin resistance, leuconostocs are intrinsically resistant to teicoplanin, fosfomycin and fusidic acid [16, 17, 26]. The vancomycin/teicoplanin resistance mechanism is known and it results from the use of D-lactate instead of D-ala in the synthesis of peptidoglycan [20]. Based on our findings, we consider that the intrinsic fosfomycin resistance in *Leuconostoc* is due to the Asp in MurA position 115 (*E. coli* numbering) known to confer resistance in *E. coli* [24]. In addition to leuconostocs, *Fructobacillus*, *Weissella*, *Oenococcus*, *Pediococcus* and lactobacilli appear to carry the resistant genotype, whereas other LAB genera have the sensitive genotype. Our findings related to fusidic acid resistance suggests that it results from point mutations within the *fusA* gene [26]. *Leuconostoc, Fructobacillus* and *Convivina* have the resistant genotype associated with a Q at the position 457 (*Staphylococcus* numbering), whereas *Weissella* and *Oenococcus* have the sensitive genotype related to a H at position 457. Recently [30], *IsaA* gene similar to a clindamycin resistant phenotype was detected in *Leuconostoc fallax* ATCC 700006^T^ and *Leuconostoc pseudomesenteroides* NCDO 768^T^. However, we did not detect this gene in the 47 *L. gelidum/ L. gasicomitatum* genomes analysed.

A form of spoilage manifested by the formation of slime has been associated with *L. gelidum*/*L. gasicomitatum* [5]. Since all strains contained genes encoding at least one glucansucrase and half of them carried also genes encoding a fructansucrase, it can be concluded that these species have a general potential to form dextran if sucrose is available as the precursor and favourable growth condition exists. On the other hand, we did not find clear evidence of ability of forming biogenic amines (BAs) that result from decarboxylation or iminase reactions related to amino acids. BAs are unwanted metabolites in foods since they are associated with health hazards and a malodour [31]. Decarboxylation of histidine as histamine leads to a food poisoning resembling a severe allergic reaction after consumption of spoiled histidine-rich food such as tuna and some fish belonging to *Scromboidaceae*. Tyramine produced from tyrosine is known to trigger migraine, whereas the polyamines putrescine, cadaverine and agmatine are considered as indicators of food spoilage. We searched the genomes but did not detect genes related to agmatine demininase or histidine or tyrosine decarboxylases. We detected a gene that is annotated as an orn/lys/arg-decaboxylase by some annotations (not NCBI) in strains AMKR21, C220d, HS9, JPBL22, Kg1-2 & Vvan9). However, this gene is only half of the length of the characterised orn/lys/arg-decaboxylases. Thus, it is more likely an unknown aminotransferase. Based on the genomic data, these leuconostocs are unlikely to produce any BAs. The ability of leuconostocs to form BAs has been reported time to time but it is probably related to the former classification of “leuconostocs” that are currently considered as *Oenococcus*, *Weissella* and *Fructobacillus.* Due to the many changes in the taxonomy of leuconostocs, one should be cautious while reading older literature and not to mix properties of these genera in these with the current leuconostocs.

## Conclusions

Genome features of 47 strains belonging to *L. gasicomitatum* and *L. gelidum* subspecies *aenigmaticum* and *gelidum* showed only limited variation even though the strains selected for this study originated from different sources, represented variable MLST and genotyping groups, and were isolated in four different countries over a time span of more than 25 years. The genetic relatedness indices as well as the phylogenetic analyses revealed that the strains formerly designated as *L. gelidum* subsp. *gasicomitatum* are not a subspecies of *L. gelidum.* Thus, the proposal of Rahkila et al. [3] to reclassify *L. gasicomitatum* as *L. gelidum* subsp. *gasicomitatum* should be rejected and the species status for *L. gasicomitatum* restored as also recently suggested by Wu and Gu [4]. Analyses of the genomes did not reveal any source attribution or other clustering associated with the lifestyle of the strains. The pangenome of *L. gasicomitatum* was estimated to include 3,046 genes of which were 1,313 (43.1%) were considered the core genes present in 99% of the assemblies. After 22 genomes, less than 15 genes were added to the pool per new genome, suggesting that this pangenome was closing slowly, whereas *L. gelidum* pangenomes (2,703 genes) remained open. According to our analyses on the 47 *L. gelidum/ L. gasicomitatum* genomes, these strains did not carry any acquired antimicrobial resistance genes or genes associated with production of harmful biogenic amines.

## Methods

### Selection and sequencing of the strains

Thirty-seven *L. gelidum* strains isolated during previous research activities from various foods and food-related samples (Table 1) were selected for genome sequencing. The source and county of origin are listed in Table 1.

For sequencing, DNA was extracted from cells collected from MRS broth cultures (25 °C) as described earlier [3]. DNA purity was confirmed using a NanoDrop 2000™ spectrophotometer and quantified using a Qubit® 3.0 fluorometer (Thermo Scientific, USA). Library preparation and sequencing were carried out at the Institute for Molecular Medicine Finland (FIMM), HiLIFE, University of Helsinki, Finland. First, a paired-end DNA library for Illumina sequencing was prepared and normalised with ~ 300 bp inserts using a Nextera XT DNA library Preparation Kit (Illumina, CA, USA). The prepared library was sequenced using an Illumina HiSeq 2500 platform for 100 bp paired-end reads.

In addition to 37 sequenced strains, three complete and seven draft genomes of *L. gelidum/L. gasicomitatum*, including the type assemblies of each subspecies, were retrieved (Supplementary Table S2). Among them are four *L. gasicomitatum* strains sequenced by Andreevskaya et al. [32] that at the time of writing were incorrectly classified as *Leuconostoc inhae* by the European Nucleotide Archive (ENA). Furthermore, the genome sequence for *Leuconostoc kimchii* IMSNU 11154^ T^ (BioProject PRJNA40837) to was used as a root in the phylogenetic analyses. All software were run with default parameters, unless otherwise stated.

### Genome assembly

Following sequencing, the quality control of the raw sequence reads was conducted with FastQC v.0.11.9 available at: http://www.bioinformatics.babraham.ac.uk/projects/fastqc/, the adaptor removal and trimming were performed with Trimmomatic v. 0.40 [33]. The draft genomes were assembled with Velvet v.1.2.08 [34] together with VelvetOptimiser v.2.2.6 (https://github.com/tseemann/VelvetOptimiser) and their quality for completeness and contamination was evaluated using CheckM v.1.0.10 [35]. The assembled genomes were annotated using the NCBI Prokaryotic Genome Annotation Pipeline (PGAP) [36].

The accession numbers and details of the genomes used in this study are provided in Supplementary Tables S1 and S2.

To obtain genome-to-genome relatedness indices, Pairwise average nucleotide identity (ANI) values based on BLAST were determined using pyani.py v.0.2.7 (https://github.com/widdowquinn/pyani) and digital DNA–DNA hybridisation (dDDH) with the genome-to-genome distance calculator 2.1 (GGDC) server (available at: http://ggdc.dsmz.de/ggdc.php) using formula 2 recommended for draft genomes. The species boundary for ANI and dDDH values were set for/at? 95 ~ 96 and 70%, respectively.

### Pangenome and core genome

The pangenome and core genome sizes were estimated using BPGA v.1.3.0 [37].

### Phylogenetic analysis

The nucleotide sequences of all 1,141 single-copy orthologous genes shared by all 47 of the genomes were retrieved by homologous searches using GET_HOMOLOGUES v.3.2.4 [38] and aligned using Prank v.140603 [39] with the “-codon” option for codon aware alignment. Gblocks v.0.91b [40] was used to select conserved regions for the construction of concatenated nucleotide sequences. An approximative Maximum-Likelihood phylogenetic tree was built using FastTree v.2.1.11 [41], with the generalised time-reversible (GTR) model*.* Maximum-Likelihood phylogenetic trees were constructed for concatenated housekeeping genes atpA, pheS and rpoA using IQ-TREE v.1.6.12 [42] with the TPM2u + F + I and the HKY + F + G4 nucleotide substitution models, respectively.

### Prediction of antibiotic resistance

The Resistance Gene Identifier (RGI) tool of the Comprehensive Antibiotic Resistance Database “CARD” version 3.1.3 [43] was used to predict resistomes by using contigs file with the parameters “Perfect, Strict, complete genes only” as search criteria. Literature information on intrinsic antibiotic resistance in *Leuconostoc* species was also collected. The protein sequences were aligned using multiple sequence alignment MAFFT v.7.408 [44]. Aligned sequences were visualized with GeneDoc v.2.7 [45].

## Supplementary Information

**Additional file 1:** **Supplementary Fig. 1.** Multiple alignment ofrepresentative MurA protein sequences from different genera of LAB, *Escherichia coli* and *Staphylococcus aureus*. The MurA sequences used in the alignment are: *S.aureus* Q931H5, *E. coli* P0A749, *Leuconostoc gasicomitatum* A0A7H9BCM4, *Fructobacillus fructosus* A0A3F3I000, *Oenococcus oeni* A0NKT6, *Weissella confusa* A0A1T4J435, *Pediococcus acidilactici* A0A1A5VP62, *Dellaglioa algida* A0A0R1HHE0, *Carnobacterium maltaromaticum* K8E679, *Enterococcus faecium* A0A133CSM8, *Lactococcus lactis* Q9CIP4 and *Streptococcus pyogenes* P0DC46. **Supplementary Fig. 2.** Multiple alignment ofrepresentative FusA protein sequences from different genera of LAB and from *Staphylococcus aureus*: The FusA sequences used in the alignment are: *Leuconostoc gasicomitatum* A0A175CRG2, *Fructobacillus *sp. A0A0J5PAW1, *Weissella viridescens *A0A0R2H6R8, *Ooenococcus oeni* Q04ED6, *S. aureus* P68790, *Lactococcus carnosus* A0A0D6E076, *Carnobacterium divergens* A0A4R9CM82, *Eenterococcus faecalis*Q839G9, *Vagococcus fessus* A0A430A960and *Paussilactobacillus oligofermentans*A0A0R1RP22.

**Additional file 2:** **Table S1.** Strains and genomes studied. **Table S2.** Accession numbers and general genome features of the 37 *L. gelium* strains sequenced in this study. **Table S3.** Accession numbers and general genome features of the 10 *L. gelium* genomes retrieved from NCBI. **Table S4.** Matrix with genome-to-genome ANI (lower part) and dDDH (upper part) values (%) obtained for 47 *L. gelidum*/*L. gasicomitatum* genomes. The values of and above 95% and 70% for ANI and dDDHA, respectively, are highlighted in gray. **Table S5.** Genes unique to *L. gelidum* subsp. *aenigmaticum* strains. **Table S6.** Genes unique to *L. gasicomitatum* strains. **Table S7.** Genes unique to *L. gelidum* subsp. *gelidum*/*aenigmaticum* strains. References to publications cited in the Supplementary **Table S1**.

## Abbreviations

AMR: Antimicrobial resistance
ANI: Pairwise average nucleotide identity
dDDH: Digital DNA–DNA hybridization
MLST: Multilocus sequence typing
PFGE: Pulsed field gel electrophoresis
RGI: Resistance Gene Identifier

## Declarations

### Availability of data and materials

The datasets generated and/or analysed during the current study are available in the NCBI nucleotide database and the accession numbers are listed in the Supplementary Tables S2 and S3.
