# Supplementary material for: Pangenome and genomic taxonomy analyses of Leuconostoc gelidum and Leuconostoc gasicomitatum
Source: BMC Genomics. 2022 Dec 9;23:818. doi: 10.1186/s12864-022-09032-3 (PMC9733070; doi:10.1186/s12864-022-09032-3)
Supplement: Supplementary file 1 — Additional file 1: Supplementary Fig. 1. Multiple alignment ofrepresentative MurA protein sequences from different genera of LAB, Escherichia coli and Staphylococcus aureus. The MurA sequences used in the alignment are: S.aureus Q931H5, E. coli P0A749, Leuconostoc gasicomitatum A0A7H9BCM4, Fructobacillus fructosus A0A3F3I000, Oenococcus oeni A0NKT6, Weissella confusa A0A1T4J435, Pediococcus acidilactici A0A1A5VP62, Dellaglioa algida A0A0R1HHE0, Carnobacterium maltaromaticum K8E679, Enterococcus faecium A0A133CSM8, Lactococcus lactis Q9CIP4 and Streptococcus pyogenes P0DC46. Supplementary Fig. 2. Multiple alignment ofrepresentative FusA protein sequences from different genera of LAB and from Staphylococcus aureus: The FusA sequences used in the alignment are: Leuconostoc gasicomitatum A0A175CRG2, Fructobacillus sp. A0A0J5PAW1, Weissella viridescens A0A0R2H6R8, Ooenococcus oeni Q04ED6, S. aureus P68790, Lactococcus carnosus A0A0D6E076, Carnobacterium divergens A0A4R9CM82, Eenterococcus faecalisQ839G9, Vagococcus fessus A0A430A960and Paussilactobacillus oligofermentansA0A0R1RP22. [file 12864_2022_9032_MOESM1_ESM.docx]

* 20 * 40 * 60 * 80 * 100 * 120 * 140 * 160 *
*S. aureus* : MDKIVIKGGNKLTGEVKVEGAKNAVLPILTASLLASDKPSKLVNVPALSDVETINNVLTTLNADV--TYKKDENAVVVDATKTLNEEAPYEYVSKMRASILVMGPLLARLGHAIVALPGGCAIGSRPIEQHIKGFEALGAEIHLENGNIYANA-KDGLKGTSIHLDFPSVGA : 169
*E. coli* : MDKFRVQGPTKLQGEVTISGAKNAALPILFAALLA-EEPVEIQNVPKLKDVDTSMKLLSQLGAKV-----ERNGSVHIDARDVNVFCAPYDLVKTMRASIWALGPLVARFGQGQVSLPGGCTIGARPVDLHISGLEQLGATIKLEEGYVKASV-DGRLKGAHIVMDKVSVGA : 165
*L. gasicomitatum* : MAKIIVHGGKRLHGEVTIGGAKNSTVALIPAAILA-DTPVKFDTVPQILDVKNLQLILNAMNVHS--VF--ANGNLDIDPTQIRESELPSGAIKSLRASYYFMGALLGRFNRATVTFPGGDNLGRRPIDQHIKGFEALGATVTESNDIIHIDATAHGLIGARIALDTVSVGA : 167
*F. fructosus* : MAKIIINGGHKVAGQVTIGGAKNSTVALIPAAILA-DTPVNFDNVPQILDVKNLQYILAAMNVHS--NF--HDGDLSIDPTTIREAPLPATAIKSLRASYYFMGALLGRFGRATVTFPGGDNLGSRPIDQHIKGFEALGAKVTEENDVIHIDATENGLKGARIALDTVSVGA : 167
*O. oeni* : MTKILINGGKRLHGEVMIGGAKNSTVALIPASILA-ETPVRFDTVPAILDVKNLQLILRSMNVSS--NF--ENGFLEIDPTHIVDAPLPKDAIKSLRASYYFMGALLGKFGKATVTFPGGDNIGPRPIDQHIKGFKALGAMVREENDTIYITTGPEGLHGAEIFFDKVSVGA : 167
*W. confusa* : MKKLVIHGGRRLSGEVTVGGAKNSTVALIPAAILA-ETPVKFDSVPDILDVYNLQVILESMNVKS--TF--FGGELMIDPTEIIEAELPSTAIKSLRASYYFMGSLLGRFGRATVTFPGGDNIGPRPIDQHIKGFEALGATVREENDTVYITA-ENGLKGARIFLDMVSVGA : 166
*P. acidilactici* : MKKMIIKGGQRLTGEVSIGGAKNSTVALIPAAILA-DTPVRFDSVPDILDVHNLMIILESMNVHS--TF--KDGVMEIDPTEIVENPLPSKAIKSLRASYYFMGSLLGRFKRSTVTFPGGDNIGPRPIDQHIKGFKALGANVTEDEDTVYISAYERGLHGAHIFLDVVSVGA : 167
*D. algida* : MKKMIIRGGKKLSGEVIIGGAKNSTVALIPAAILA-DTPVHFDSVPDILDVHNLMKILDQMNVKS--EF--NEGNLTVDPTMIKDEPLPGGAIKSLRASYYFMGALLGRFGRAIVGFPGGDNIGPRPIDQHIKGFKALGAEVIEEDDAVIITSGPEGLKGARIFMDLVSVGA : 167
*C. maltaromaticum* : MKKLIVNGGRPLSGEITISGAKNSTVALIPAAILA-DSPVTLEGVPDIQDVHSLIEILEIMNVKV--DF--DGSTLVIDPTKIVSIPMPSGKIKSLRASYYFMGALLSKFGQGVVGLPGGCFLGPRPIDQHLKGFEALGATVDNELGAMYLRSGDEGLTGARVYLDVVSIGA : 167
*E. faecium* : MKKIVIKGNRPLAGEVTISGAKNSAVALIPAAILA-DSPVILEGVPDIQDVHSLIEILEIMGATV--HF--SNNILEIDPREVVSIPMPHGKINSLRASYYFMGTLLGKFGEAVVGLPGGCYLGPRPIDLHVKGFEALGAEVTNEHGAMYLRTENKGLRGNRIFMDVVSVGA : 167
*L. lactis* : MKKIVINGGKRISGTIPISGAKNSVVALIPATILA-NDVVTLEGVPDISDVASLVEIMEIMGAKIERNL--EEGRLVIDTRSVVSRPLPYGKINSLRASYYFNGALLGRFGQATVGLPGGCDLGPRPTDLHDKAFKALGAKKIQEEEAEHLEVIGDSLVGTTIYMDVVSVGA : 169
*S. pyogenes* : MRKIIINGGKALSGEVAVSGAKNSVVALIPAIILA-DDIVILDGVPAISDVDSLIEIMELMGATV--NY--HGDTLEIDPRGVQDIPMPYGKINSLRASYYFYGSLLGRFGQAVVGLPGGCDLGPRPIDLHLKAFEAMGVEVSYEGENMNLSTNGQKIHGAHIYMDTVSVGA : 167


 180 * 200 * 220 * 240 * 260 * 280 * 300 * 320 * 340
*S. aureus* : TQNIIMAASLAKGKTLIENAAKEPEIVDLANYINEMGGRITGAGTDTITINGVESLHGVE-HAIIPDRIEAGTLLIAGAITRGDIFVRGAIKEHMASLVYKLEEMGVELDYQEDGIRVR-AEGELQPVDIKTLPHPGFPTDMQSQMMALLLTANGHKVVTETVFENRFMHVA : 339
*E. coli* : TVTIMCAATLAEGTTIIENAAREPEIVDTANFLITLGAKISGQGTDRIVIEGVERLGGGV-YRVLPDRIETGTFLVAAAISRGKIICRNAQPDTLDAVLAKLRDAGADIEVGEDWISLDMHGKRPKAVNVRTAPHPAFPTDMQAQFTLLNLVAEGTGFITETVFENRFMHVP : 336
*L. gasicomitatum* : TINIILAAVRAKGQTVIENAAREPEIIDIATFLNNMGANVRGAGTDTIRIAGVPSLKAKNTHTVIPDRIEAGTYMSMAAAFGDGVTIKNVIPEHLESYTSKLIEMGVNLDIGEDMIHVY-KSDDLKPVAIKTMPYPGFATDLQQPITPLLLMANGESTIQDTIYPKRVKHIA : 338
*F. fructosus* : TINIILAAVRAEGQTIIENVAKEPEIIDIATFLNNMGANIRGAGTDTIRITGVDTLKAQNTHSVIPDRIEAGTYMSLAAAFGDGVTIQNVIPEHLESYTSKLMEMGVDMQIGDESIYIG-PSRNLKPVSIKTMPYPGFATDLQQPITPLLFLAEGESTIIETIYPKRIRHIA : 338
*O. oeni* : TMNLIMASVRAKGLTILENVAREPEIIDLATFLNNMGANIRGAGTDTIRITGVPVLRSSAVHAIIPDRIEAGTYITAAAAIGDGVTVKNVIPEHLESFTSKLIEMGVHLRISEDEIYVP-ATSHLKPVTITTAPFPGFATDLQQPISPLLMKADGVSTIVDTIYPQRIRHIV : 338
*W. confusa* : TMNIILAAVHAEGTTILENAAREPEIIDLATFLNNMGAQIRGAGTDVIRITGVPKLESKNTHTIIPDRIEAGTYLALAAAQGDGVLVKNIIPEHLESFTAKMIEMGVDLDVREDSIFVP-KVENLKPIHIKTAPFPGFATDLQQPITPLLTLAEGESIISETIYPERTRHIP : 337
*P. acidilactici* : TINVILAAVKAQGVTTIENAAREPEIIDLAMFLNNMGAKIRGAGTDVIRIEGVDKLVSTATHTIIPDRIEAGTYLSLAAAVGDGILVNNIIPEHLESFTSKMIELGVDLKIDGDKIYVP-KVEKMRGIEVNTNPFPGFATDLQQPLTASLLKAQGRSVVNDHIYPERVKHVS : 338
D. algida : TINILLAAVRAKGTTVIENAAKEPEIIDIATFLNNMGAHIRGAGTGEIRIQGVDSLRAVNTHTIIPDRIEAGTYLSLAAAMGEGVLVKNVITEHLDSFLAKLSEMGVTFKVNEDSIFVP-GGDEIIPIEVTTSPYPGFATDLQQPITAVMIKAAGESRIVDTLYPERIKHIP : 338
C. maltaromaticum : TINLMLAAVRAKGKTIIENAAREPEIIDVATLLNNMGAKVRGAGTDIIRIEGVTELKGCR-HTIIPDRIEAGTYLSMAAAMGTDVVVKNVIFEHLEGLIAKMEEMGVPMEIGEDSIRVL-EAKNLKMVSIKTLPYPGFATDLQQPLTPLLLKAHGEGMIVDTIYPKRVKHIP : 337
*E. faecium* : TINVMLAAVKAKGQTVIENAAREPEIIDVATLLNNMGAKVRGAGTDVIRIEGVETLHGCR-HFMIPDRIEAGTYLALAAAVGNGIKVKNVIFEHLESFIAKLQEIGVHMKISEDEIEVY-PSKELKPANIMTYPYPGFATDLQQPLTALLLMTTGTSEIIDTIYAKRVNHVP : 337
*L. lactis* : TINTMLAASRAKGLTIIENAAREPEIIDVATLINNMGAQVRGAGTDIIRITGVDEMHGAQ-HTVIPDRIEAGTYLALAAAMGDGVIIENVIYEHLESFIAKLEEMGVGLTIREDSIEVH-KSENLKSVNITSVPYPGFATDLQQPITPLLLKAKGRGSIVDTIYQKRVNHVP : 339
*S. pyogenes* : TINTMVAATKAQGKTVIENAAREPEIIDVATLLNNMGAHIRGAGTDIITIQGVQKLHGTR-HQVIPDRIEAGTYIALAAAIGKGVKITNVLYEHLESFIAKLEEMGVRMTVEEDAIFVE-KQESLKAITIKTSPYPGFATDLQQPLTPLLLKADGRGTIIDTIYEKRINHVP : 337


 * 360 * 380 * 400 * 420 *
*S. aureus* : EFKRMNANINVEG-RSAKLEGKSQLQGAQVKATDLRAAAALILAGLVADGKTSVTELTHLDRGYVDLHGKLKQLGADIERIND------- : 421
*E. coli* : ELSRMGAHAEIES-NTVICHGVEKLSGAQVMATDLRASASLVLAGCIAEGTTVVDRIYHIDRGYERIEDKLRALGANIERVKGE------ : 419
*L. gasicomitatum* : ELRRMGGDVDSEKPGEIKINYSPRLVGTDVQAAEIRAGAALIIAAIMAEGETVINDADHILRGYDRIQDKLTHLGAEISIEGIDLINLMP : 428
*F. fructosus* : ELRRMGADVSSLNPGEIKVNHSERLVGTTVEAAEIRAGAALVIAAVMADGQTVIDNASHILRGYDRIAEKLRGLGVDLKIEGVDLINLMP : 428
*O. oeni* : ELRRMGGDISSLEGGVIQVNESPRLVGTDVQAAEIRAGAALVIAGLMADGQTIIDHAEHILRGYEQIQQKLTGLGASVSVLGVDIPNLMP : 428
W. confusa : ELQKLGVAIENPEYGVITVANSNNFHGASVAAAEIRAGAALVTAGLMADGITEVTNAEHVLRGYDHIIHKLTMLNADIQIAED------- : 420
*P. acidilactici* : ELQKMGANIQHAD-GVIYVDYTDQLYGANVEAGEIRAGACLMIAAFMADGTTTITKADNILRGYDSLVKKLTKLGADVEITADQSL---- : 423
*D. algida* : EMQRMGVKIESKD-GVIRINQSTELTGQTVYADEIRAGAALLEVALMTNGTTVIEKAENILRGYDCVVKKMTNLSADVEIIDED------ : 421
C. maltaromaticum : ELVRMGAKARVET-DMILLEGPTKLKGVEVEASDLRAGACLVTAGLMAEGTTTITGVENILRGYDHIVEKLTALGADIKMIEVEDK---- : 422
*E. faecium* : ELARMGADITVEG-NMIIVNGPNKLHGTEVVASDLRAGACLVIAGLLAEGTTTIYNVDYILRGYDHIIEKLTALGASIEMIEEVGAE--- : 423
*L. lactis* : ELARMGANISVLD-DRIIYDAPNELTGSCVQATDLRAGAALVTAGIIASGTTKISNIEFILRGYDHIIEKLTAVGVDIQLIEE------- : 421
*S. pyogenes* : ELMRMGADISVIG-GQIVYQGPSRLTGAQVKATDLRAGAALVTAGLMAEGKTEITNIEFILRGYASIIAKLTALGADIQLIED------- : 419

Supplementary Fig. 1.

* 20 * 40 * 60 * 80 * 100 * 120 * 140
*L. gasicomitatum* : MA-KREYPLERTRNIGIMAHIDAGKTTTTERILYYTGKIHKIGETHDGASQMDFMEQEKERGITIQSAATTAVWHGFFDQFEKTPYRVNIIDTPGHVDFTIEVERALRVLDGAVAVLDGAAGVEPQTETVWRQATTYDVPRIV : 142
*Fructobacillus* sp. : MA-KREYPLDHTRNIGIMAHIDAGKTTTTERILYYTGKIHKIGETHDGASQMDFMEQEKERGITIQSAATTAVWRGFFDQYEKNPFRVNIIDTPGHVDFTIEVERALRVLDGAVAVLDGAAGVEPQTETVWHQATTYNVPRIV : 142
*W. viridescens* : MANKREYPLNRTRNIGIMAHIDAGKTTTTERILYYTGKIHKIGETHDGASQMDFMDQEKERGITIQSAATTAVWHGFHDQYKNDPFRINIIDTPGHVDFTIEVERSLRVLDGAVAVLDGSAGVEPQTETVWRQAETYDVPRIV : 143
*O. oeni* : MD-TREFPLDRTRNIGIMAHIDAGKTTTTERILYYTGKIHKIGETHDGASQMDFMDQEKERGITIQSAATTAIWHGFHEQWKDTPYRVNIIDTPGHVDFTIEVERSLRVLDGAIAVLDGAAGVEPQTETVWRQATTYAVPRLV : 142
*S. aureus* : MA--REFSLEKTRNIGIMAHIDAGKTTTTERILYYTGRIHKIGETHEGASQMDWMEQEQDRGITITSAATTAAWEG---------HRVNIIDTPGHVDFTVEVERSLRVLDGAVTVLDAQSGVEPQTETVWRQATTYGVPRIV : 132
*L. carnosus* : MA--REFSLENTRNIGIMAHVDAGKTTTTERVLYYTGKIHKIGETHEGASQMDWMAQEQERGITITSAATTAAWHD---------TRINIIDTPGHVDFTIEVQRSLRVLDGAVTVLDAQSGVEPQTETVWRQATEYGVPRIV : 132
*C. divergens* : MA-NREFSLEKTRNIGIMAHVDAGKTTTTERILYYTGKIHKIGETHEGASQMDWMEQEQERGITITSAATTAEWKN---------YRVNIIDTPGHVDFTIEVQRSLRVLDGAVTVLDSQSGVEPQTETVWRQATDYKVPRIV : 133
E. faecalis : MA--REFSLEKTRNIGIMAHVDAGKTTTTERILYYTGKIHKIGETHEGASQMDWMEQEQERGITITSAATTAQWKG---------YRVNIIDTPGHVDFTIEVQRSLRVLDGAVTVLDSQSGVEPQTETVWRQATEYKVPRIV : 132
*V. fessus* : MP--REFTLDKTRNIGIMAHIDAGKTTTTERILYYTGKIHKIGETHDGASQMDWMEQEQERGITITSAATTASWKG---------HRVNIIDTPGHVDFTVEVERSLRVLDGAVTVLDAQSGVEPQTETVWRQATTYGVPRTV : 132
*P. oligofermentans* : MANKREFPLEMTRNIGIMAHIDAGKTTTTERILYYTGKIHKIGETHDGASQMDWMAQEQERGITITSAATTAAWKD---------HRINIIDTPGHVDFTIEVERSLRVLDGAVAVLDAQAGVEPQTETVWRQASEYNVPRIV : 134


 * 160 * 180 * 200 * 220 * 240 * 260 * 280
*L. gasicomitatum* : FVNKMDKMGADFQMSVDSIHERLQVNAEAIQWPIGAEDDFEAVIDLITQEAYYPVDELGEKWEARDIPAELKDLAEEKRNTLIEAVADVDDDLMEKYLEGEDISVEELKAAVRRATLALQFYPVLAGSAYKDKGVQMMLDAVV : 285
*Fructobacillus* sp. : FVNKMDKLGADFEMSVNSMHERLQVNAEAIQWPIGAEDDFEGVIDLIEEKAYWPTDDLGSKWEPREIPADYQDIVSTKRETLIEAVSDVDEELMEKYLEGEEISIDELKAAIRRATLNLEFYPVLAGSAYKDKGVQMMLDAVV : 285
*W. viridescens* : FVNKMDKMGADFGMSVDSLKDRLDANAKAVQWPIGAEDDFAGIIDLITKEAWYPTTELGEEWEVRDMPADYADLVEEKYNELVEAIADVDDEIMDKYLGGEEISADELKAAIRRATLNLEFYPVFAGSAYKDKGVQMVLDGVV : 286
*O. oeni* : FVNKMDKMGADFQMSVDSLKERLDVNAKAIQWPIGAEDDFAGVIDLIQREAWYPDDKLGTEWEKRPIPDDLKDLVEEKRDELIEAVADVDDSLMEKYLGEEEITVDDLKAAIRRATLALKFYPVLAGSAYKDKGVQLLLDAVV : 285
*S. aureus* : FVNKMDKLGANFEYSVSTLHDRLQANAAPIQLPIGAEDEFEAIIDLVEMKCFKYTNDLGTEIEEIEIPEDHLDRAEEARASLIEAVAETSDELMEKYLGDEEISVSELKEAIRQATTNVEFYPVLCGTAFKNKGVQLMLDAVI : 275
*L. carnosus* : FANKMDKIGADFFYSLNTLHDRLNANAHPIQIPIGAEEDFEGIIDLVRMRAEVYTNDLGTDILDEEIPEEYLAQAEEWRAKLIEAVADTDEDIMMKYLEGEEITEAELKAAIRKATINVEFYPMLAGSAFKNKGVQMMLDAVV : 275
*C. divergens* : FCNKMDKIGADFLYSVNSLHERLQANAHPIQLPIGAEDDFTGIIDLVKMKAEIYTNDLGTDIQETDIPEEYVEAATEWRKKLIEAVVETDEELMMKYLDGEEITEEELKAGIRQATINVEFFPVMAGSAFKNKGVQLMLDAVL : 276
*E. faecalis* : FCNKMDKIGADFFYSVESLHDRLQANAHPIQIPIGAEEDFTGIIDLIKMKAEIYTNDLGTDIQETDIPEDYLEKAQEWREKLVEAVAETDEDLMMKYLEGEEITEEELVAGIRQATINVEFFPVLAGSAFKNKGVQLMLDAVL : 275
*V. fessus* : FINKMDKIGADFLYSVSTLHDRLQANAVPVQLPIGAEDEFTGIIDLITMKAEMYTNDLGTDIREEEIPAEYLELATEWREKLVEAVAETDEELTMKYLEGEEISIEELNAAIRRATINVELFPVYCGSAFKNKGVQLMLDGVI : 275
*P. oligofermentans* : FANKMDKMGANFDFSVETIKDRLQANALPIQMPIGAEDDFRGIIDLVEMKADLYHGDDGAEWDTVDIPEEFAEEAKKRHDAMVETLADIDDGIMEKYLNGEEVSKAEIKAAIRKGTLELTLFPVLAGSAFKNKGVQMMLDAVV : 277


 * 300 * 320 * 340 * 360 * 380 * 400 * 420
*L. gasicomitatum* : DYLPGPLDVKAYI---ANDPKTGEEIDLIADDSKSFAALAFKIMTDPFVGRLTFMRVYTGTLQSGSYVQNTSSDTRERVGRLLQMHATSRTEIEEVFSGDIAAAIGLKNTTTGDSLTSVDHQLILESMEFPEPVIELAIEPKT : 425
*Fructobacillus* sp. : DYLPGPLEVRPYV---ATDPKTDEEVDLVADDSKPFAALAFKIMTDPFVGRLTFMRVYTGTLQSGSYVQNTSSDTRERVGRLLQMHATSRTEIEEVFSGDIAAAIGLKNTTTGDSLTAVDHPLILESMEFPEPVIELAIEPKT : 425
W. viridescens : DYLPSPLEVKPYV---ATDPKTGEEVDLTANDEDPFAALAFKVMTDPFVGRLTFLRVYTGSLEAGSYVMNTSRDKRERVGRLLQMHATSRTEIPEVFSGDIAAAIGLKATTTGDSLTAPDRPLVLESMEFPDPVIQLAIEPAT : 426
*O. oeni* : DYLPSPLEVRPYT---ATDPDTGDEVDLKADDKKPFAALAFKIMTDPYVGRLTFLRVYTGTLKSGSYVQNTTKDTRERVGRLLQMHAITRREIDEVFSGDIAAAIGLKATSTGDSLTSVDRPLVLESMEFPDPVIQMAVEPKT : 425
*S. aureus* : DYLPSPLDVKPIIGHRASNP--EEEVIAKADDSAEFAALAFKVMTDPYVGKLTFFRVYSGTMTSGSYVKNSTKGKRERVGRLLQMHANSRQEIDTVYSGDIAAAVGLKDTGTGDTLCGEKNDIILESMEFPEPVIHLSVEPKS : 416
*L. carnosus* : DYLPAPTDVPAIK---GVTPD-GEETERHADDSEPFAALAFKVMTDPFVGRLTFFRVYSGVLSSGSYVLNASKGKRERIGRILQMHANTRNEINDVFSGDIAAAVGLKDTTTGDSLTDEKNPVILESIEFPEPVIQVMVEPKS : 414
*C. div*ergens : DYLPSPLDVDAIK---GIDVKTEEETTRPADDSAPFASLAFKVMTDPFVGRLTFFRVYSGVLESGSYVLNASKNKKERVGRILQMHANTRKEIDKVYSGDIAAAVGLKDTTTGDTLCALEAPVILESIEFPEPVIQVAVEPKS : 416
*E. faecalis* : DYLPSPLDIDAIK---GIDTKTDEETTRPADDEAPFASLAFKVMTDPFVGRLTFFRVYSGVLESGSYVLNASKGKKERIGRILQMHANTRQEIDKVYSGDIAAAVGLKDTTTGDTLCALDAPVILESIEFPDPVIQVAVEPKS : 415
*V. fessus* : DYLPAPTDVAAIT---GINPDTDEEVVVPSTDDAPFAALAFKVMTDPFVGRLTFFRVYSGVLQSGSYVKNASKGKRERVGRILQMHANSREEISEVYAGDIAAAVGLKDTTTGDTLCDEKNLVILESMEFPEPVIEVAIEPKS : 415
*P. oligofermentans* : DYLPSPLDVRPYN---ATDPDTDENVELEADDTKPFAALAFKIATDPFVGRLTFIRVYTGTLESGSYVLNTTKGKRERVGRLLQMHSNHRQEIPEVFSGDIAAAIGLKNTTTGDSLTSPDHPLKLESMEFPDPVIQVAVEPKS : 417


 * 440 * 460 * 480 * 500 * 520 * 540 * 560 *
*L. gasicomitatum* : KADQDKLSNAIQKLAEEDPSFRATTNPETGDTLIAGMGELQLDIMVDRMRREFNVEATVGAPQVAYREAFTKT-VQARGYFKRQSGGKGQYGDVYIEFSPNEEGAGFEFDDAIVGGVVPREYIPSVEAGLKDSLNAGPLAGFP : 567
*Fructobacillus* sp. : KADQDKLATALQKLAEEDPSFRATTNQETGDTLIAGMGELQLDIMVDRMKREFNVEATVGAPQVAYREAFTKT-VQARGFFKRQSGGKGQYGDVWIEFSPNEEGAGFEFEDAIVGGVVPREYIPSVEAGLKDALNAGPLAGFP : 567
*W. viridescens* : KADQDKMSNALQKLAEEDPSFRAETNEETGDTLISGMGELHLDIIVDRLKREFGVDANVGAPQVAYREAFTKT-VQARGYFKRQSGGKGQYGDVWIEFSPNEEGAGFEFEDAIVGGAVPREYIPSVEAGLRDSMNAGPIAGFP : 568
*O. oeni* : KADQEKMAEALQKLSEEDPSFHAETNSETGQTLISGMGELHLDIMVDRMRREFNVDVNVGTPQVAYREAFTKT-VQAQGKYIRQSGGKGQYGDVWIEFSPNDEGKGFEFDNAIVGGAVPREYIPAVEQGLKEAMQSGPLAGYP : 567
*S. aureus* : KADQDKMTQALVKLQEEDPTFHAHTDEETGQVIIGGMGELHLDILVDRMKKEFNVECNVGAPMVSYRETFKSS-AQVQGKFSRQSGGRGQYGDVHIEFTPNETGAGFEFENAIVGGVVPREYIPSVEAGLKDAMENGVLAGYP : 558
*L. carnosus* : KADQDKMGVALQKLAEEDPSFRVETNVETGETLIAGMGELHLDILVDRMRREFNVDANVGAPQVSYRETFRAPVTQAEGKFVRQSGGKGQYGHVWVEFTPNEEGKGFEFENAIVGGVVPREYIPAVEKGLAEAMQNGVLAGYP : 557
*C. divergens* : KADQDKMGIALQKLAEEDPSFRVETNAETGETVISGMGELHLDVLVDRMRREFNVDASVGAPQVSYRETFRGS-TKAEGKFVRQSGGKGQYGHVWIEFTPNEEGAGFEFENAIVGGVVPREYIPAVKAGLEGSLDNGVLAGYP : 558
*E. faecalis* : KADQDKMGVALQKLAEEDPSFRVETNVETGETVISGMGELHLDVLVDRMKREFKVEANVGAPQVSYRETFRAA-TKAEGKFVRQSGGKGQYGHVWVEFTPNEEGKGFEFENAIVGGVVPREYIPAVEKGLEDSMNNGVLAGYP : 557
*V. fessus* : KADQDKMGTALQKLSEEDPTFRASTNPETGETIIAGMGELHLDVLVDRMRREFKVEANVGAPQVSYRETFRAPVTQAEGKFVRQSGGKGQYGHVWVEFTPNEEGAGFAFENAIVGGVVPREYIPAVEAGLRASMDNGVLAGYP : 558
*P. oligofermentans* : KADQDKMDIALQKLAEEDPSFKAETNPETGETLIAGMGELHLDIIIDRMRREFNVEATVGAPQVAYREAFTKQ-TSAQGKFVRQSGGKGQYGDVSIEFTPNEEGAGFEFEDAIVGGVVPREYIPSVEQGLRESLENGVLAGYP : 559


 580 * 600 * 620 * 640 * 660 * 680 * 700 *
*L. gasicom*itatum : LVDLKAKLYDGSYHDVDSSEAAFKIAASLALREAAKTAGAVILEPIMAVDIVAPEDNLGDVMGHVSARRGMIEGQESRGPVLAVKAKVPLSEMFGYATTLRSATQGRGTFQMVFDHYEAVPKNIQEEIIKNSGKED--- : 703
*Fructobacillus* sp. : LVDLKAKLYDGSYHDVDSSEAAFKIAASLALKEASKTAGAVILEPIMAVDIVVPEENLGDVMGHVSARRGLIEGQENRGPVLAVKAQVPLSEMFGYATTLRSATQGRGTFQMVFDHYQAVPKNVQEEIIKAQGKEA--- : 703
*W. viridescens* : LVDLKAKLYDGSYHDVDSSEAAFKIAASLALREAAKTAGAVILEPIMRVDITVPEDNLGDVMGHVSARRGLLEGQEQRGNSMIIHAKVPLSEMFGYATTLRSSTQGRGTFQMQFDHYEAVPKNVQDEIVKKYGKHD-ED : 706
*O. oeni* : LVDLKAKLYDGSYHEVDSSEAAFKIAASMSLREASKTAGAVILEPIMKVSIVSPIDNLGDVMGHVSARRGMIEDQETHGNTVTVTSKIPLAEMFGYTTTLRSATQGRGTFQMFFDHYEAVPRNVQEDIIKNAGKE---- : 702
*S. aureus* : LIDVKAKLYDGSYHDVDSSEMAFKIAASLALKEAAKKCDPVILEPMMKVTIEMPEEYMGDIMGDVTSRRGRVDGMEPRGNAQVVNAYVPLSEMFGYATSLRSNTQGRGTYTMYFDHYAEVPKSIAEDIIKKNKGE---- : 693
*L. carnosus* : LVDVKAKLYDGSYHDVDSNETAFRVAASYALKAAAPKAKPVILEPMMKVTVTVPEENLGDIMGHVTARRGRVEGMEAHGNSQIVHAFVPLAEMFGYATTLRSSTQGRGTFMMVFDHYEDVPKSVQEAIIKKNQG----- : 691
*C. divergens* : LVDIKAKLYDGSYHDVDSNETAFKVAASMALKAAAKKANPVILEPMMKVIVTVPEDYLGDIMGHITARRGRVEGMEAHGNSQIVNAIVPLANMFGYATTLRSSTQGRGTFMMVFDHYEDLPKSIQEEIISKNGGN---- : 693
*E. faecalis* : LVDIKAKLYDGSYHDVDSNETAFRVAASMALKAAAKNANPVILEPMMKVTITVPEDYLGDIMGHVTSRRGRVEGMEAHGNSQIVNAMVPLAEMFGYATTLRSATQGRGTFMMVFDHYEDVPKSVQEEIIKKNGGNA--- : 693
*V. fess*us : LVDIKAKLYDGSYHDVDSNETAFRVAASMALKAAAKKAQPAILEPIMGIEIVIPEDYLGDIMGHVTSRRGRVEGMEARGNSQVVRAMIPLAEMFGYATTLRSATQGRGVFTMTFDHYEDVPKSVQEEIIKKNGGSN--- : 694
*P. oligofermentans* : LVDLKAKLYDGSYHDVDSSEASFKVAASLALRNAAKNAGAVILEPIMKVDIVIPEEYMGDIMGQVTARRGRIDGMEERGNAQQVHSYVPLSEMFGYATTLRSASQGRGVFTMTFDHYEAVPKSVQEEIIKKNGGSNIKE : 698

Supplementary Fig.2.

Legends to the supplementary figures

Supplementary Fig. 1. Multiple alignment of representative MurA protein sequences from different genera of LAB, *Escherichia coli* and *Staphylococcus aureus*. The MurA sequences used in the alignment are: *S. aureus* Q931H5, *E. coli* P0A749, *Leuconostoc gasicomitatum* A0A7H9BCM4, *Fructobacillus fructosus* A0A3F3I000, *Oenococcus oeni* A0NKT6, *Weissella confusa* A0A1T4J435, *Pediococcus acidilactici* A0A1A5VP62, *Dellaglioa algida* A0A0R1HHE0, *Carnobacterium maltaromaticum* K8E679, *Enterococcus faecium* A0A133CSM8, *Lactococcus lactis* Q9CIP4 and *Streptococcus pyogenes* P0DC46.

Supplementary Fig. 2. Multiple alignment of representative FusA protein sequences from different genera of LAB and from *Staphylococcus aureus*: The FusA sequences used in the alignment are: *Leuconostoc gasicomitatum* A0A175CRG2, *Fructobacillus* sp. A0A0J5PAW1, *Weissella viridescens* A0A0R2H6R8, *Ooenococcus oeni* Q04ED6, *S. aureus* P68790, Lactococcus carnosus A0A0D6E076, *Carnobacterium divergens* A0A4R9CM82, *Eenterococcusfaecalis* Q839G9, *Vagococcus fessus* A0A430A960 and *Paussilactobacillus oligofermentans* A0A0R1RP22
